# NAADP-mediated Ca^2+^ signaling promotes autophagy and protects against LPS-induced liver injury

**DOI:** 10.1096/fj.201601290R

**Published:** 2017-04-06

**Authors:** So-Young Rah, Young-Hoon Lee, Uh-Hyun Kim

**Affiliations:** * Department of Biochemistry, Chonbuk National University Medical School;; † National Creative Research Laboratory for Ca^2+^ Signaling Network, Chonbuk National University Medical School;; ‡ Department of Oral Anatomy, School of Dentistry, Chonbuk National University, Jeonju, South Korea;; § Institute of Cardiovascular Research, Chonbuk National University, Jeonju, South Korea

**Keywords:** calcium, CD38, TFEB, gene expression, hepatocytes

## Abstract

LPS has been shown to induce hepatocyte autophagy, but little is known about how LPS is able to do this during acute toxic liver injury. Our aim was to determine the existence of any selective Ca^2+^ signaling coupling to hepatocyte autophagy in response to LPS. LPS increased the autophagic process in hepatocytes, and CD38 knockdown prevented this response. Ned19, a specific inhibitor for nicotinic acid adenine dinucleotide phosphate (NAADP), prevented LPS-mediated Ca^2+^ signaling and autophagosome formation in hepatocytes. CD38 overexpression protected the liver from LPS/d-galactosamine (GalN)-induced injury, and NAADP administration promoted autophagosome formation and protected hepatocytes from injury induced by LPS/GalN. Autophagy was promoted by the up-regulation of autophagy-related gene expression *via* NAADP-mediated Ca^2+^ signaling in response to LPS. However, CD38-knockout mice displayed down-regulation in hepatocyte gene expression. Ned19 also inhibited the NAADP-stimulated induction of gene expression by inhibiting the LPS-induced nuclear translocation of transcription factor EB (TFEB). Hepatocyte autophagy protects against LPS-induced liver injury *via* the CD38/NAADP/Ca^2+^/TFEB pathway. The role of NAADP-mediated Ca^2+^ signaling in the autophagic process will help elucidate the complexities of autophagy regulation, which is essential toward the discovery of new therapeutic tools against acute liver injury.—Rah, S.-Y., Lee, Y.-H., Kim, U.-H. NAADP-mediated Ca^2+^ signaling promotes autophagy and protects against LPS-induced liver injury.

Autophagy is a major intracellular degradation system activated in response to the deprivation of nutrients or growth factors (1). Autophagy processes consist of autophagosome formation, maturation and fusion with lysosomes (referred to autophagic flux), and subsequent breakdown and release of macromolecules back into the cytosol (2, 3). The autophagic flux is a multistep pathway, with each step characterized by a transition of the intracellular autophagosomes to autophagolysosomes (4). Recent studies have identified the connections between autophagic flux and pathologic conditions (5, 6).

The molecular mechanisms or regulatory pathways for autophagic flux have been extensively studied since the discovery of mammalian autophagy (*Atg*) genes. More than 30 *Atg* genes have been shown to participate in autophagic flux–related processes (7). Among them, mammalian microtubule-associated protein 1 light chain 3 (LC3), which is a homolog of yeast Atg8, is widely used as a marker for monitoring the autophagy process (8). After its synthesis, LC3 proteins are specifically cleaved at their C termini by the ATG4 proteases, producing a cleaved form called LC3-I (9). Upon autophagy induction, LC3-I is conjugated to phosphatidylethanolamine. The resulting phosphatidylethanolamine-conjugated form II of ATG8 proteins, called LC3-II, is tightly bound to the autophagosomal membranes and serves as an autophagic marker protein (10, 11).

Intracellular Ca^2+^ mobilization is one of the key regulators of autophagy (12, 13). Autophagosomes trafficking and fusing with lysosomes have been shown to be Ca^2+^-dependent events (14). In particular, lysosomal Ca^2+^ signaling controls the activity of transcription factor EB (TFEB), a master transcriptional regulator of lysosomal biogenesis and autophagy, through calcineurin (15). CD38 is a multifunctional enzyme responsible for the production of nicotinic adenine acid dinucleotide phosphate (NAADP) and cyclic ADP-ribose (cADPR) (16). In addition to the inositol trisphosphate pathway, NAADP and cADPR have been shown to mobilize Ca^2+^ *via* distinct mechanisms. NAADP is one of the most potent endogenous Ca^2+^-mobilizing messengers. NAADP regulates Ca^2+^ release from a lysosome-related Ca^2+^ stores, leading to global [Ca^2+^] increase in the cytosol (17, 18). Although NAADP-sensitive lysosome–derived Ca^2+^ participates in the physiologic regulation of cell functions in a variety of cells (19–21), the role of this process in autophagy has not been thoroughly explored, particularly in relation to pathologic diseases.

Activation of autophagy is known to ameliorate liver pathologic conditions such as fatty liver disease (22). Although the mechanism is not clear, hepatocyte autophagy confers resistance to LPS-induced liver injury (23). Because NAADP-mediated Ca^2+^ mobilization is associated with autophagic flux (24), we investigated the role of CD38 and NAADP/Ca^2+^ signaling in autophagic responses in the liver. Cd38^+/+^ and Cd38^−/−^ mice were administered LPS after d-galactosamine (GalN) to cause liver injury. CD38 knockdown and treatment with Ned19, a specific inhibitor of NAADP-mediated Ca^2+^ signals, resulted in impaired autophagosome formation and autophagy-related gene induction in hepatocytes upon LPS/GalN stimulation. These results unveil the potential roles of CD38/NAADP for protecting the liver from inflammation by promoting autophagy. A comprehensive knowledge on the regulatory role of NAADP-mediated Ca^2+^ in the autophagic process holds promise for developing a novel therapeutic strategy for acute liver injury.

## MATERIALS AND METHODS

### Antibodies and reagents

Antibodies were obtained as follows: LC3B (2775; Cell Signaling, Danvers, MA, USA), p62 (5114; Cell Signaling), ATG5 (12994; Cell Signaling), ATG7 (8558; Cell Signaling), Bcl-2 (BS1511; Bioworld Technology, St. Louis Park, MN, USA), Caspase-3 (9662; Cell Signaling), Caspase-9 (9504; Cell Signaling), TFEB (MBS9125929; MyBioSource, San Diego, CA, USA or A303-673A; Bethyl Laboratories, Montgomery, TX, USA), CD38 (sc-7049; Santa Cruz Biotechnology, Dallas, TX, USA), GAPDH (sc-166574; Santa Cruz Biotechnology), Actin (MAB1501; Millipore, Billerica, MA, USA). Ned19 and xestospongin C were from Santa Cruz Biotechnology (Santa Cruz Biotechnology). 8-Bromo-ADP-ribose was from the Biolog Life Science Institute (Bremen, Germany). NAADP/acetoxymethyl ester (membrane-permeant NAADP analog) was from AAT Bioquest (Sunnyvale, CA, USA). All other reagents were obtained from Sigma-Aldrich (St. Louis, MO, USA).

### Animals and treatments

Cd38^−/−^ mice (B6.129P2-Cd38^tm/Lud^) were purchased from The Jackson Laboratory (Bar Harbor, ME, USA). Mice were bred and housed in the facilities at the Chonbuk National University Medical School under specific-pathogen–free conditions. All animal-based investigations were designed and performed in accordance with the *Guide for the Care and Use of Laboratory Animals* [National Institutes of Health (NIH), Bethesda, MD, USA; Pub. No. 85-23, revised 1996]. The entire project was reviewed and approved by the Institutional Animal Care and Use committee of the Chonbuk National University Medical School (CBU 2014-00031). To establish acute liver injury, mice were injected intraperitoneally with LPS (2 μg/kg) and galactosamine (700 mg/kg) (25).

### Serum alanine transaminase and aspartate aminotransferase analysis

Serum alanine aminotransferase (ALT) and aspartate aminotransferase (AST) were measured using a diagnostic kit (Asan Pharmaceutical Co., Seoul, South Korea) following the manufacturer’s instructions.

### Adenovirus preparation

Adenovirus-containing mouse Cd38 was purchased from Vector BioLabs (Malvern, PA, USA). Recombinant adenovirus for *in vivo* study was purified by CsCl_2_ density gradient centrifugation. Adenovirus expressing LacZ (Ad-LacZ) was used as control. Virus (1 × 10^9^ pfu) was intravenously administered to mice, and then CD38 expression was confirmed by Western blotting in liver homogenates from the mice.

### Primary hepatocyte culture

Primary hepatocytes were isolated from 6- to 8-wk-old male C57BL/6J mice as previously described (26). The inferior vena cava was cannulated and perfused at 4 ml/min. The portal vein was sectioned to allow flow through the liver. The liver was first perfused with HBSS containing 0.5 mM EGTA and 10 mM Hepes at pH 7.4, followed by perfusion with collagenase solution (0.5 mg/mouse Liberase; Roche, Mannheim, Germany) in Williams’ Medium E (Thermo Fisher Scientific, Waltham, MA, USA) for 3 min at 4 ml/min. The liver was dissected, and hepatocytes were isolated by mechanical dissection, filtered through a sterile 70-μm filter, and washed twice by centrifugation at 50 *g* for 2 min each. The hepatocytes were cultured in Primaria plates (BD Biosciences, Mississauga, ON, Canada) with Williams’ Medium E containing 1% l-glutamine and penicillin/streptomycin and supplemented with 10% fetal bovine serum (Thermo Fisher Scientific). Four hours after plating, the medium was replaced with fresh medium.

### Calcium measurements

Changes of [Ca^2+^]_i_ in hepatocytes were determined as described previously (26). Hepatocytes were incubated in Williams’ Medium E (phenol red free; 5% fetal bovine serum) containing 5 μM Fluo-4 AM (Thermo Fisher Scientific) at 37°C for 40 min. Cells were washed 3 times with this medium, and changes in [Ca^2+^]_i_ were determined at 488 nm excitation/530 nm emission by an air-cooled argon laser system. The emitted fluorescence at 530 nm was collected using a photomultiplier. The image was scanned using a confocal microscope (Nikon, Tokyo, Japan).

### Measurement of intracellular NAADP concentration

Intracellular NAADP concentration (NAADP_i_) was measured using a cyclic enzymatic assay as described previously (27). Briefly, cells were treated with perchloric acid, and precipitates were removed by centrifugation. Perchloric acid was removed by mixing the aqueous sample with a solution containing 3 volumes of 2 M KHCO_3_ and vortexed. The resulting KClO_4_ precipitate was removed by centrifugation at 20,000 *g* for 10 min. The supernatant was adjusted to pH 8.0. To remove all contaminating nucleotides, the samples were incubated overnight with the following hydrolytic enzymes at 37°C: 2.5 units/ml apyrase, 0.125 unit/ml NADase, 2 mM MgCl_2_, 1 mM NaF, 0.1 mM PP_i_, and 0.16 mg/ml NMN-AT in 20 mM sodium phosphate buffer (pH 8.0). Enzymes were removed by filtration using Centricon-3 filters (Millipore). After the hydrolytic treatment, alkaline phosphate (10 units/ml) was added overnight at 37°C to convert NAADP to NAAD. The alkaline phosphate was removed by filtration using Centricon-3 filters. The samples were further incubated with the cycling reagent (30 μl) containing 2% ethanol, 100 μg/ml alcohol dehydrogenase, 20 μM resazurin, 10 μg/ml diaphorase, 10 μM riboflavin 5′-phosphate, 10 mM nicotinamide, 0.1 mg/ml bovine serum albumin (BSA), and 100 mM sodium phosphate (pH 8.0) at room temperature for 4 h. An increase in the resorufin fluorescence was measured at 544 nm excitation and 590 nm emission using a fluorescence plate reader (Spectra-Max Gemini; Molecular Devices Corp.). Various known concentrations of NAADP were also included in the cycling reaction to generate a standard curve.

### Real-time quantitative PCR

Total RNA was isolated from tissues or cells using the RNeasy Mini Kit (Qiagen, Valencia, CA, USA). cDNA was synthesized by reverse transcription from 50 ng total RNA using a cDNA Reverse Transcriptase Kit (TaKaRa, Tokyo, Japan). Real-time quantitative PCR was carried out in a 384-well plate using the ABI Prism 7900HT Sequence Detection System (Thermo Fisher Scientific) with the following conditions: 95°C, 10 min (95°C, 10 s; 60°C, 10 s; 72°C, 15 s) for 40 cycles. Real-time quantitative PCR results were normalized against an internal control GAPDH. The sequences of primers used in study are listed in Supplemental Table 1.

### Immunoblotting

Cells and tissues were lysed in RIPA buffer (50 mM Tris-HCl, pH 7.4, 150 mM NaCl, 1% sodium deoxycholate, 0.1% SDS, and 1% triton X-100 supplemented with phosphatase and protease inhibitor cocktail) (Roche). Lysates were centrifuged at 15,000 *g* for 15 min at 4°C, and the supernatant was collected. Protein concentration was measured using a BCA protein assay kit (Thermo Fisher Scientific). Lysates were boiled in Laemmli sample buffer for 5 min, separated by SDS-PAGE, transferred to polyvinylidene fluoride membranes, and probed with primary antibodies (1:2000). After incubation with secondary antibodies conjugated with horseradish peroxidase (1:2000; Cell Signaling), chemiluminescence was detected using LAS1000 systems (Fujifilm, Tokyo, Japan). Immunoblot densitometric quantification was performed using ImageJ (NIH).

### Microscopy for autophagy

Primary hepatocytes were seeded in collagen-coated confocal dishes and transfected with mCherry-green fluorescent protein (GFP)-LC3 plasmid. The mCherry-GFP-LC3 plasmid encoding the tandem fluorescent reporter was generated in the laboratory of Dr. Terje Johansen (University of Tromsø, Tromso, Norway) and obtained from (plasmid 22418; Addgene, Cambridge, MA, USA) using Lipofectamine 2000 (Thermo Fisher Scientific) for 24 h. Nuclei were stained with DAPI. Autophagosomes and autophagolysosomes were visualized with a confocal microscope (Nikon) and counted using the Granularity plug-in module of the MetaMorph software package (Molecular Devices, Sunnyvale, CA, USA).

### Confocal imaging

For TFEB immunostaining, cells were grown on collagen-coated confocal dishes, fixed with ice-cold methanol for 10 min, and washed with ice-cold PBS. After blocking with 3% BSA, 0.25% Triton X-100, and PBS at room temperature for 1 h, samples were incubated with anti-TFEB mAb (1:100) (MyBioSource) followed by Alexa Fluor 546–conjugated donkey anti-rabbit antibody (1:200; Thermo Fisher Scientific) in the presence of 1% BSA at room temperature for 1 h. Nuclei were stained with DAPI. Cells were visualized with a Zeiss LSM510 Axiovert 200M laser-scanning confocal microscope. The images were obtained using ×63 Zeiss Plan-Aprochromat objective and LSM510 (v.7.1).

### Electron microscopy

Liver tissues were fixed with a fixation solution (2% paraformaldehyde and 2% glutaradehyde in 0.05 M sodium cacodylate buffer, pH 7.2) after mice were perfused with cold PBS. Fixed samples were embedded in Spurr’s resin (14300; Electron Microscopy Sciences, Hatfield, PA, USA), and thin sections (80 nm) were cut and processed for transmission electron microscopy. After dehydration, thin sections were stained with uranyl acetate and lead citrate and observed under a JEM 1011CX electron microscope with accelerating voltage of 100 kV (H7650; Hitachi, Tokyo, Japan). Images were acquired from a randomly selected pool of 5–8 fields under each condition.

### Histology and immunohistological staining

Mouse livers were fixed in 4% paraformaldehyde. Paraffin-embedded liver sections were used for hematoxylin and eosin (H&E) staining. TUNEL assay was performed with the DeadEnd Colorimetric System kit (Promega, Madison, WI, USA) according to the manufacturer’s instructions.

### Statistical analysis

Data are represented as the means ± sd of at least 3 independent experiments. Statistical analysis was performed using Student's *t* test or ANOVA as appropriate. A value of *P* < 0.05 was considered significant.

## RESULTS

### Autophagy formation is impaired in Cd38^−/−^ liver with hepatic cell death upon LPS/GalN administration

**Figure 1.**
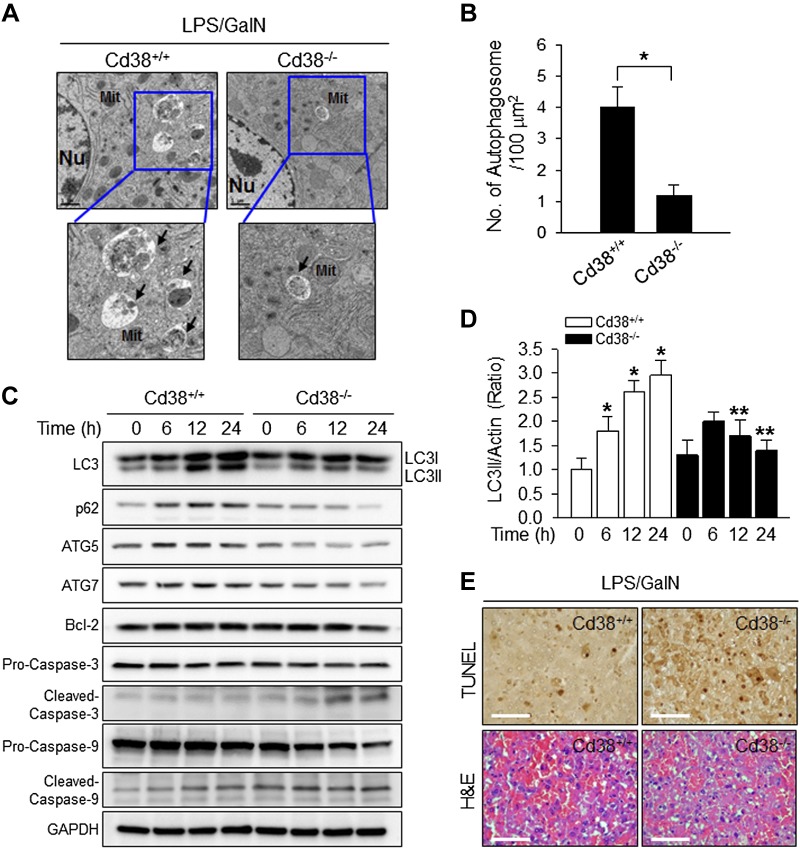
CD38 deficiency impairs autophagy formation in LPS/GalN-induced acute liver injury. *A*) Cd38^+/+^ and Cd38^−/−^ mice were intraperitoneally injected with LPS (2 μg/kg) and GalN (700 mg/kg; *n* = 5). After 6 h, the livers were fixed, sectioned, and stained with uranyl acetate and lead citrate. Autophagosomes were examined by transmission electron microscopy. Arrows indicate autophagosomes. Mit, mitochondria; Nu, nucleus. Magnification, ×1 μm. *B*) The number of autophagosomes was quantified for each experiment (*n* = 3) under the conditions described in *A*. Data are means ± sd. *P < 0.001. *C*) CD38 is involved in LPS-induced autophagy of hepatocytes. Primary hepatocytes prepared from Cd38^+/+^ and Cd38^−/−^ mice were treated with 100 ng/ml LPS for the indicated time. Cells were lysed, and immunoblotting was performed to determine LC3 (autophagy marker), p62, ATG5, ATG7, Bcl-2, Caspase-3, Caspase-9, and GAPDH. *D*) The plot shows the means ± sd from 3 independent experiments. *P < 0.001 *vs*. control, **P < 0.01, Cd38^+/+^ *vs*. Cd38^−/−^. *E*) Comparison of liver apoptosis in Cd38^+/+^ and Cd38^−/−^ mice 6 h after treatment with 2 μg/kg LPS and 700 mg/kg GalN using TUNEL staining and H&E stain. Scale bars, 50 μm.

CD38 is known to be associated with the autophagic process in coronary arterial myocytes of mice fed the high-fat Western diet (6). In this study, we investigated the role of CD38 in autophagy processing by comparing the histology of livers from Cd38^+/+^ and Cd38*^−/−^* mice challenged with LPS and GalN for 6 h to induce acute injury. Examination of hepatic cells by transmission electron microscopy showed that Cd38*^+/+^* hepatocytes had many autophagosomes around the damaged mitochondria, compared with Cd38*^−/−^* hepatocytes, which had few autophagosomes in response to LPS/GalN (**Fig. 1*A***). Numbers of autophagosomes plotted in a histogram clearly indicated that the number of autophagosomes in Cd38*^−/−^* hepatocytes was significantly less than that in Cd38*^+/+^* hepatocytes (Fig. 1*B*). These results demonstrate the importance of CD38 for the regulation of autophagic flux in hepatocytes *in vivo* upon LPS/GalN administration.

We compared the levels of LC3-I and LC3-II that are widely used as markers for autophagic processing (5) in the hepatocytes from Cd38*^+/+^* and Cd38*^−/−^* mice after LPS stimulation (Fig. 1*C*). The intensities of the bands, which were normalized against actin as internal controls, were compared in a histogram (Fig. 1*D*). The results showed that higher levels of both LC3-I and LC3-II were induced by LPS in the Cd38*^+/+^* hepatocytes than those in Cd38*^−/−^* hepatocytes, supporting the functional connection between CD38 and autophagy. Because p62 serves as a link between LC3 and ubiquitinated substrates and because ATG5 and ATG7 act as components of the autophagosome, we included p62, ATG5, and ATG7 in our Western blot analysis to monitor autophagic flux (28). Similar to LC3, LPS induced a significantly higher level of p62, ATG5, and ATG7 in Cd38*^+/+^* hepatocytes than in Cd38*^−/−^* hepatocytes (Fig. 1*C*, *D*).

Lack of autophagy affects apoptosis *via* caspase activation (29). Therefore, we examined apoptosis in the hepatocytes from Cd38*^+/+^* and Cd38*^−/−^* mice after LPS stimulation. The levels of cleaved caspase-3 and caspase-9 were significantly higher in Cd38*^−/−^* hepatocytes than in Cd38*^+/+^* hepatocytes, and Bcl-2 (antiapoptosis protein) expression was significantly reduced in Cd38*^−/−^* hepatocytes (Fig. 1*C*). Furthermore, we examined whether CD38 could rescue apoptotic cell death induced by LPS/GalN using a TUNEL assay. The number of apoptotic cells was significantly higher in Cd38*^−/−^* mice than in Cd38*^+/+^* hepatocytes (Fig. 1*E*). These results indicate that CD38 protects the liver from acute injury induced by LPS/GalN.

### Autophagosome formation and autophagy-related genes expression are impaired in Cd38^−/−^ hepatocytes

**Figure 2.**
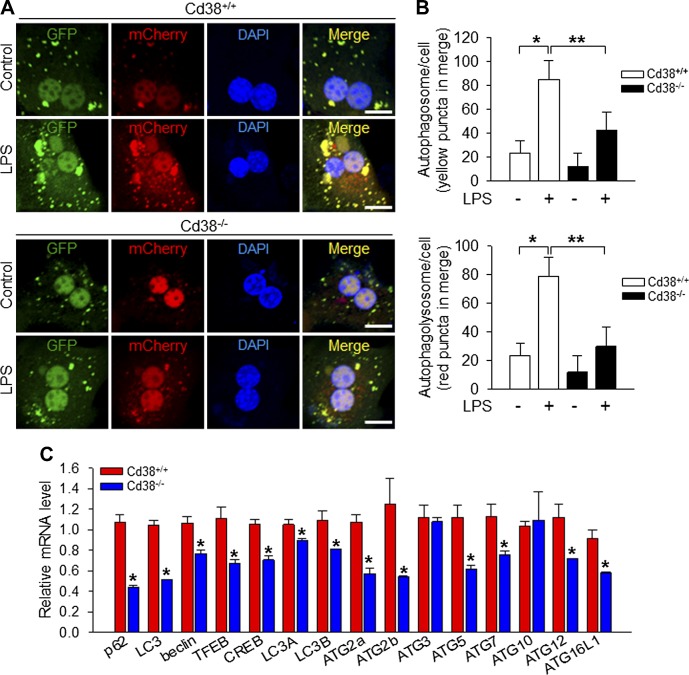
Involvement of CD38 in LPS-induced autophagosome formation and autophagy-related gene expression. *A*) LC3 puncta formation in Cd38^+/+^ and Cd38^−/−^ hepatocytes transfected with mCherry-GFP-LC3 prior to stimulation with 100 ng/ml LPS for up to 24 h. The nucleus was stained with DAPI (blue). Scale bars, 10 μm. *B*) Summarized data showing the number of autophagosomes (yellow/orange puncta) and autophagolysosomes (red puncta) in the merged image. The plot shows the means ± sd from 3 independent experiments. *P < 0.001, **P < 0.001. *C*) Expression level of mRNA of autophagy-related genes in hepatocytes of Cd38^+/+^ and Cd38^−/−^. Data are means ± sd from 3 independent experiments. *P < 0.001, Cd38^+/+^ *vs*. Cd38^−/−^.

Next we performed histologic analysis to confirm the role of CD38 in the induction of LC3 on Cd38*^+/+^* and Cd38*^−/−^* hepatocytes transfected with tandem mCherry-GFP-tagged LC3 vector. In this system, autophagosomes and autophagolysosomes are distinguished by the different colors emitted. LC3 in the autophagosomes appears as yellow colored puncta due to combined green GFP and red mCherry fluorescence. In contrast, LC3 in the autophagolysosomes emits only red fluorescence because once autophagosomes are fused with lysosomes, the green GFP fluorescence is lost due to the decay caused by acidic lysosomal pH (30). Consistent with our previous Western blot results, there were fewer LC3 puncta in autophagosomes and autophagolysosomes in Cd38*^−/−^* hepatocytes than in Cd38*^+/+^* hepatocytes after LPS treatment (**Fig. 2*A***). This is also shown in the histograms in Fig. 2*B*. These results indicate that CD38 is important for facilitating the overall autophagic process induced by LPS.

To determine whether CD38 promotes autophagy processing at the genetic level, we compared mRNA levels of various autophagy-related genes, including LC3, p62, and ATG in Cd38*^+/+^* and Cd38*^−/−^* hepatocytes (Fig. 2*C*). The results showed that the levels of mRNAs for all the autophagy-related genes we tested were significantly lower in Cd38*^−/−^* hepatocytes than in Cd38*^+/+^* hepatocytes. These results suggest that CD38 may promote autophagy processing by enhancing the expression of autophagy-related genes in hepatocytes.

### CD38 protects the liver from LPS/GalN-induced injury

**Figure 3.**
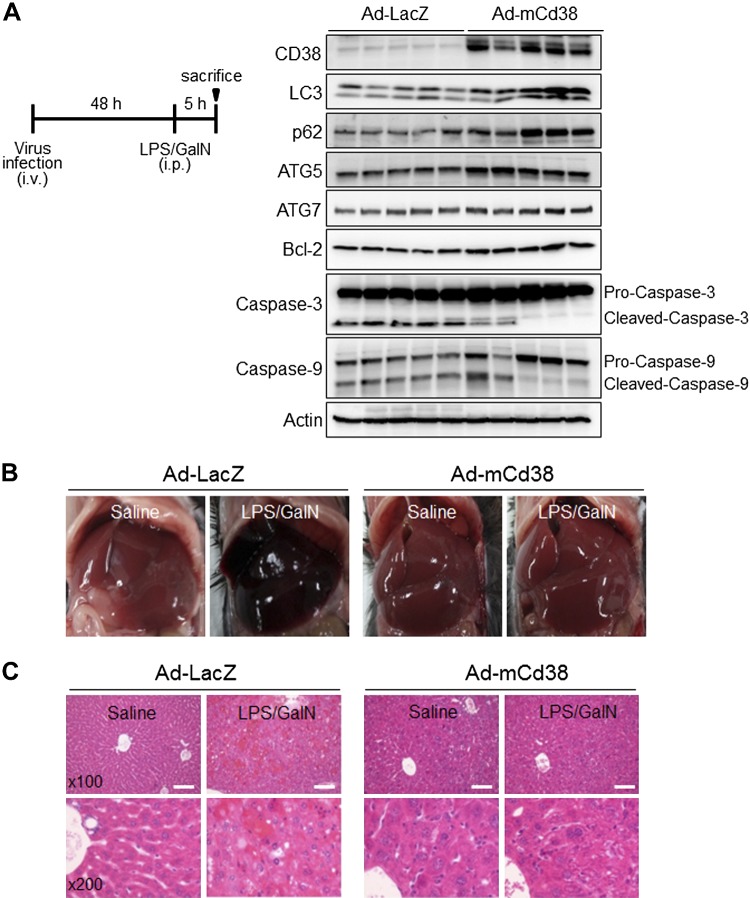
CD38 overexpression protects LPS/GalN-induced acute liver injury. *A*) Experimental schedule for LPS/GalN-induced acute liver injury. Mice were infected with adenovirus particles (1 × 10^9^ pfu) *via* tail vein 48 h prior to injection with LPS (2 μg/kg) and GalN (700 mg/kg) for 6 h (*n* = 5 for each group). CD38 expression was confirmed by immunoblotting in liver homogenates from mice infected with Ad-LacZ or Ad-mCd38 (*n* = 3 independent experiments). Immunoblotting was performed for autophagy marker proteins (LC3, p62, ATG5, and ATG7), apoptosis marker proteins (Caspase-3 and Caspase-9), and an antiapoptosis marker protein (Bcl-2). *B*) Representative liver tissue images. *C*) H&E staining. Scale bars, 100 μm.

The findings described above show the protective role of CD38 in hepatocytes at the cellular level (Fig. 1*E*). We then conducted a follow-up study to see whether CD38 protects the liver from injury induced by LPS/GalN. To this end, mice were infected with recombinant adenovirus expressing mouse Cd38 (Ad-mCd38) or Ad-LacZ as control. Hepatic tissue lysates from the mice infected with Ad-mCd38 showed markedly enhanced expression of CD38 than those from the mice infected with Ad-LacZ (**Fig. 3*A***). The mice were injected with LPS/GalN, and then the livers were isolated for pathologic evaluation.

LPS/GalN injection to the mice infected with Ad-mCd38 showed higher levels of LC3II, p62, ATG5, and ATG7, which are autophagy marker proteins, than mice infected with Ad-LacZ, whereas apoptosis was significantly reduced in the mice infected with Ad-mCd38 (Fig. 3*A*). LPS/GalN injection to mice infected with Ad-LacZ induced severe parenchymal hemorrhage in the liver. In contrast, almost no parenchymal hemorrhage and no degenerative architecture were induced in the mice infected with Ad-mCd38 (Fig. 3*B*). These results demonstrate that CD38 markedly alleviates the pathologic injury induced by LPS/GalN at the organ level. The tissues obtained from the livers were further evaluated by H&E staining. Hepatic tissues from mice infected with adenovirus expressing Cd38 look healthier compared with those from control mice infected with LacZ expressing adenovirus (Fig. 3*C*). These results suggest that CD38 protects the liver from injury induced by LPS/GalN and thereby enhances cell viability.

### NAADP promotes autophagosome formation and protects the hepatocytes from injury induced by LPS/GalN

**Figure 4.**
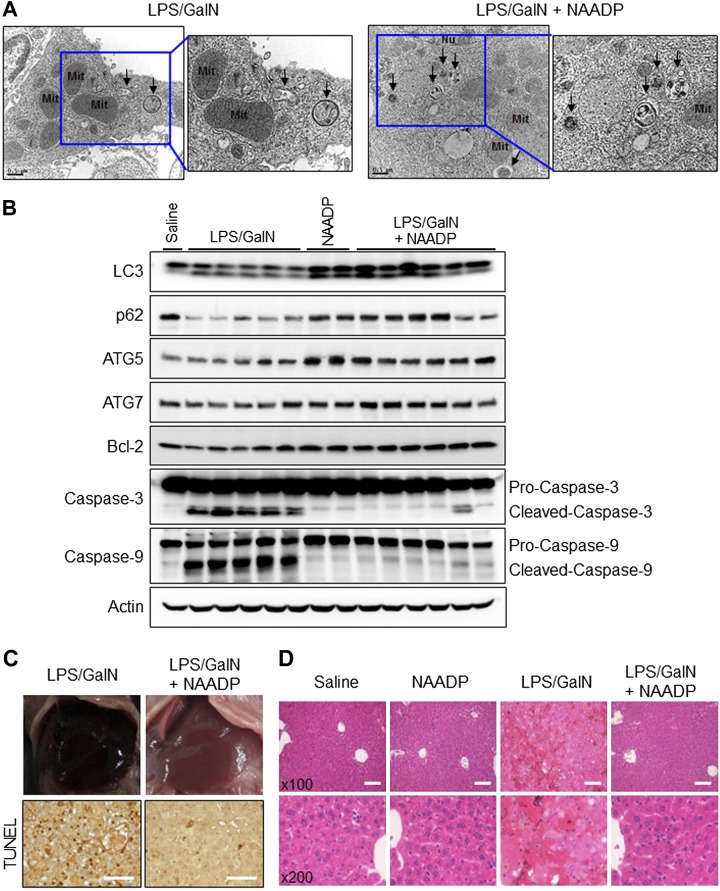
Autophagy induction by NAADP suppresses LPS/GalN-induced acute liver injury. Cd38^−/−^ mice were intraperitoneally injected with LPS (2 μg/kg) and GalN (700 mg/kg) for 6 h. NAADP (0.181 mg/kg) was injected intravenously 30 min after administration with LPS and GalN in mice (*n* = 5 or 6). *A*) The livers were fixed, sectioned, and stained with uranyl acetate and lead citrate. Autophagosomes were examined by transmission electron microscopy. Arrows indicate autophagosomes. Mit, mitochondria; Nu, nucleus. Scale bars, 0.5 μm. *B*) Immunoblotting of liver lysates was performed using anti-LC3, p62, ATG5, ATG7, Bcl-2, Caspase-3, Caspase-9, and actin antibodies (*n* = 3 independent experiments). *C*) Representative livers tissue images and TUNEL staining. Scale bars, 100 μm. *D*) H&E stain. Scale bars, 100 μm.

NAADP is a product of CD38/ADP-ribosylcyclase (20). Therefore, NAADP could exert the same effects as CD38 and promote autophagosome formation and protect the liver from injury induced by LPS/GalN. To test the role of NAADP, Cd38*^−/−^* mice were intraperitoneally injected with LPS/GalN and subsequently with NAADP or saline. Hepatocytes obtained from Cd38*^−/−^* mice treated with LPS/GalN followed by injection with or without NAADP were examined by electron microscopy (**Fig. 4*A***). The electron microscopic images showed more autophagosomes in hepatocytes from Cd38*^−/−^* mice injected with NAADP than from Cd38*^−/−^* mice injected with saline. These results were confirmed by Western blotting, which showed higher levels of LC3II, p62, ATG5, and ATG7 in hepatocytes from Cd38*^−/−^* mice treated with NAADP than those from control mice (Fig. 4*B*). Moreover, the increase of cleaved caspase-3 and caspase-9 by LPS/GalN treatment was significantly reduced in mice treated with LPS/GalN followed by injection with NAADP, and Bcl-2 (antiapoptosis protein) expression was significantly increased in these mice (Fig. 4*B*). Livers were obtained from these mice and examined for pathologic conditions induced by LPS/GalN (Fig. 4*C*). We found that the livers obtained from Cd38*^−/−^* mice injected with NAADP looked healthier than those from saline-injected Cd38*^−/−^* mice with apparent parenchymal hemorrhage and degenerative architecture. These results were confirmed by TUNEL assay. Hepatocytes from the mice injected with NAADP remained highly viable even after LPS/GalN treatment, in contrast to those from control mice with many apoptotic cells (Fig. 4*C*, lower panel). Consistent with the these observations, H&E staining of the liver tissues revealed that injection of NAADP into Cd38*^−/−^* mice ameliorated parenchymal hemorrhage induced by LPS/GalN (Fig. 4*D*). These results indicate that NAADP exerts effects similar to those by CD38 overexpression in promoting autophagy and protecting the liver from injury induced by LPS/GalN.

### Ned19, an antagonistic analog of NAADP, blocks LPS-mediated Ca^2+^ signals and autophagosome formation in hepatocytes

**Figure 5.**
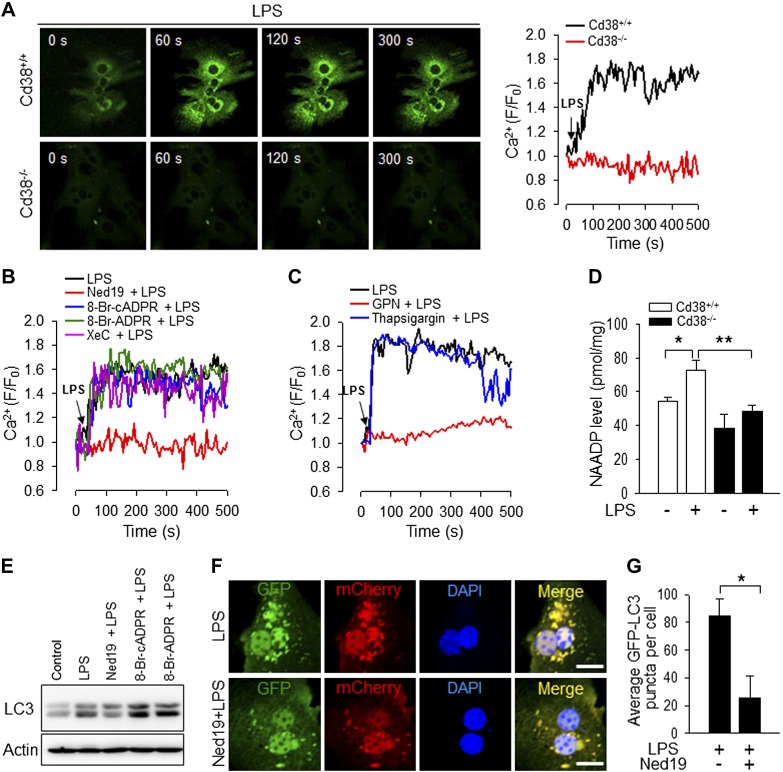
Role of NAADP-mediated Ca^2+^ signaling in LPS-induced autophagy of hepatocytes. *A*) LPS-induced Ca^2+^ increase is mediated by CD38 in hepatocytes. After loading the cells with fluo-4 AM, the change of [Ca^2+^] was measured using a confocal microscope. The arrow indicates the time point at which 100 ng/ml LPS was added. *B*) Ned19 inhibits LPS-induced [Ca^2+^] increase in hepatocytes. Ned19 (10 μM), 8-bromo-cyclic ADP-ribose (8-Br-cADPR) (100 μM), 8-bromo-ADP-ribose (8-Br-ADPR) (100 μM), or Xestospongin C (XeC) (2 μM) was preincubated for 30 min before treatment with LPS. The arrow indicates the time point at which 100 ng/ml LPS was added. *C*) LPS-induced [Ca^2+^] increase is inhibited by GPN but not by thapsigargin. GPN (50 μM) or thapsigargin (1 μM) was preincubated for 30 min before treatment with LPS. The arrow indicates the time point at which 100 ng/ml LPS was added. *D*) LPS induces NAADP production in a CD38-dependent manner. Hepatocytes from Cd38^+/+^ and Cd38^−/−^ mice were treated 100 ng/ml LPS for 5 min, and then levels of NAADP were determined using the cyclic method. Data are means ± sd from 3 independent experiments. *P < 0.001, **P < 0.05. *E*) Hepatocytes were treated with 100 ng/ml LPS for 12 h and lysed, and Western blot was performed to detect LC3 (autophagy marker) and actin. Ned19 (10 μM), 8-Br-cADPR (100 μM), or 8-Br-ADPR (100 μM) was preincubated for 30 min before the treatment with LPS (*n* = 3 independent experiments). *F*) Ned19 inhibits LPS-induced autophagosome formation in hepatocytes. Hepatocytes expressing GFP-LC3 were treated with LPS (100 ng/ml) for 24 h, and GFP-LC3 puncta were examined by confocal microscope. Ned19 (10 μM), an antagonistic analog of NAADP, was preincubated for 30 min before treatment with LPS. Nuclei were stained with DAPI (blue). Scale bars, 20 μm. *G*) GFP-LC3 puncta were quantified for each experiment. The plot shows the means ± sd from 3 independent experiments. *P < 0.001.

Although NAADP-mediated Ca^2+^ signals are known to be required for the autophagy process (24), the role of NAADP in hepatocytes has not been fully scrutinized, especially regarding the autophagy process. To determine whether LPS mobilizes Ca^2+^ and whether this requires CD38, we monitored Ca^2+^ signals in Cd38*^+/+^* and Cd38*^−/−^* hepatocytes after LPS stimulation. LPS induced Ca^2+^ signals in Cd38*^+/+^* hepatocytes but not in Cd38*^−/−^* hepatocytes, suggesting that CD38 participates in part, if not fully, in LPS-mediated Ca^2+^ signals (**Fig. 5*A***). We further analyzed the LPS-mediated Ca^2+^ signals by testing the effect of various inhibitors of Ca^2+^ mobilization, including Ned19 (an NAADP antagonist), 8-bromo-cyclic ADP-ribose (a cADPR antagonist) and 8-bromo-ADP-ribose (an ADPR antagonist), and xestospongin C (an IP_3_ receptor blocker). Interestingly, among the inhibitors we tested, Ned19 was the only inhibitor that could block the LPS-mediated Ca^2+^ signals in hepatocytes (Fig. 5*B*), indicating that NAADP is the main Ca^2+^ signaling second messenger responsible for the LPS-mediated Ca^2+^ signals in hepatocytes.

Because NAADP activity is limited to intracellular Ca^2+^ stores in the acidic organelles of certain cell types (31), we extended our study to identify the target Ca^2+^ stores for NAADP using inhibitors such as Gly-Phe-naphylamide (GPN) and thapsigargin. GPN induces osmotic lysis of the acidic compartment, and thapsigargin depletes endoplasmic reticulum Ca^2+^ stores by inhibiting the sarcoplasmic/endoplasmic reticulum Ca^2+^ ATPase (SERCA). GPN, but not thapsigargin, blocked the LPS-mediated Ca^2+^ signals (Fig. 5*C*), supporting our notion that CD38/NAADP signaling axis, whose function is largely dependent on the acidic compartment, plays a pivotal role in the LPS-mediated Ca^2+^ flux in hepatocytes. To corroborate these findings, we examined whether NAADP was produced in hepatocytes upon LPS treatment. LPS significantly increased NAADP levels in Cd38*^+/+^* hepatocytes but not in Cd38*^−/−^* hepatocytes. Moreover, basal levels of NAADP in Cd38*^−/−^* hepatocytes before treatment with LPS were significantly lower than those in Cd38*^+/+^* hepatocytes (Fig. 5*D*). This result indicates that CD38 is involved in the LPS-induced NAADP formation of primary hepatocytes.

Because Ned19 blocked the LPS-mediated Ca^2+^ mobilization, we examined whether Ned19 interferes with autophagosome formation. Assessment of LC3 levels by Western blotting showed that Ned19 markedly attenuated the LPS-induced LC3II expression (Fig. 5*E*). Furthermore, confocal images indicated that Ned19 inhibited LC3 puncta formation induced by LPS (Fig. 5*F*), as also shown in the histogram in Fig. 5*G*. Taken together, these data clearly demonstrate the strong inhibitory activity of Ned19 on the LPS-mediated autophagosome formation in hepatocytes.

### NAADP promotes autophagy process at a transcriptional level

Our results demonstrate that CD38/NAADP-dependent Ca^2+^ signaling promotes the autophagy process at the cellular and whole organ levels and that Ned19 suppresses this effect. Using a quantitative real-time PCR assay, we examined whether NAADP-AM, a cell-permeable NAADP, enhances the expression of autophagy-related genes at the transcriptional level and whether Ned19 reverses the NAADP activity.

**Figure 6.**
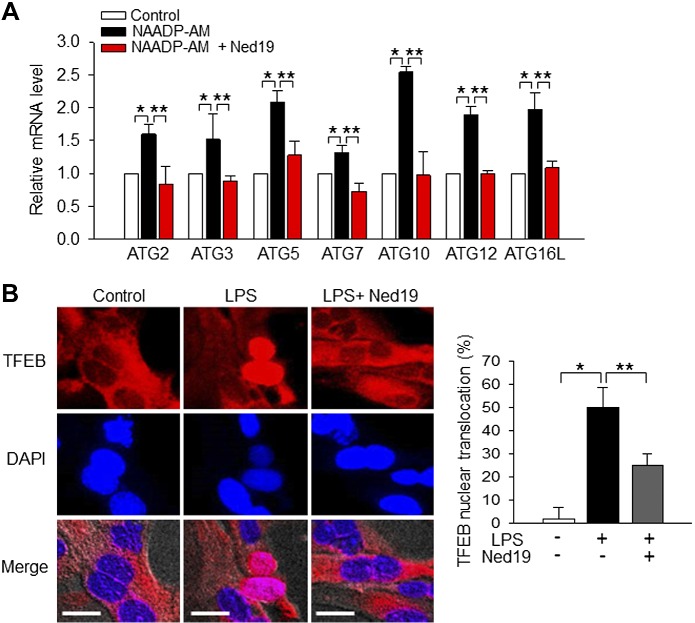
NAADP-induced nuclear translocation of TFEB regulates the transcriptional level of autophagy-related genes. *A*) Levels of mRNA were determined by real-time quantitative PCR after the treatment with NAADP-AM (50 nM) for 4 h. Ned19 (10 μM) was preincubated for 30 min before the treatment with NAADP-AM. Data are means ± sd from 3 independent experiments. *P < 0.001, ** *P* < 0.05. *B*) Hepatocytes were treated with LPS (100 ng/ml) for 6 h and stained with anti-TFEB (red), and then TFEB localization was examined by confocal microscopy. Nucleus is stained with DAPI (blue). Ned19 (10 μM), an antagonistic analog of NAADP, was preincubated for 30 min before treatment with LPS. Scale bars, 10 μm. The graph represents the percentage of TFEB nuclear translocation. Data are means ± sd from 3 independent experiments. *P < 0.001, **P < 0.05.

Like CD38, NAADP-AM markedly increased the expression of all autophagy-related genes we tested, including various ATG homologs (**Fig. 6*A***). Importantly, these enhancing effects of NAADP-AM on gene expression were greatly attenuated by Ned19. These results suggest that NAADP promotes the autophagy process at the transcriptional level.

Transcription factor EB (TFEB) is a master molecule for lysosomal biogenesis and for expression of autophagy and lysosomal genes (15). Because nuclear translocation of TFEB is required for transcription of the autophagy and lysosome related genes, we tested whether Ned19 could inhibit nuclear translocation of TFEB induced by LPS. Hepatocytes were incubated with LPS in the presence or absence of Ned19 and subsequently stained with anti-TFEB (red). TFEB nuclear localization was assessed under a confocal microscope. Nuclei were stained with DAPI (blue). Nuclear translocation of cytosolic TFEB was detected in hepatocytes in response to LPS stimulation. This TFEB translocation induced by LPS was markedly inhibited by Ned19 (Fig. 6*B*). Thus, these results suggest that NAADP promotes the autophagy process at the transcriptional level, up-regulating the expression of autophagy related genes.

### CD38 and NAADP protect the liver from injury induced by LPS/GalN

**Figure 7.**
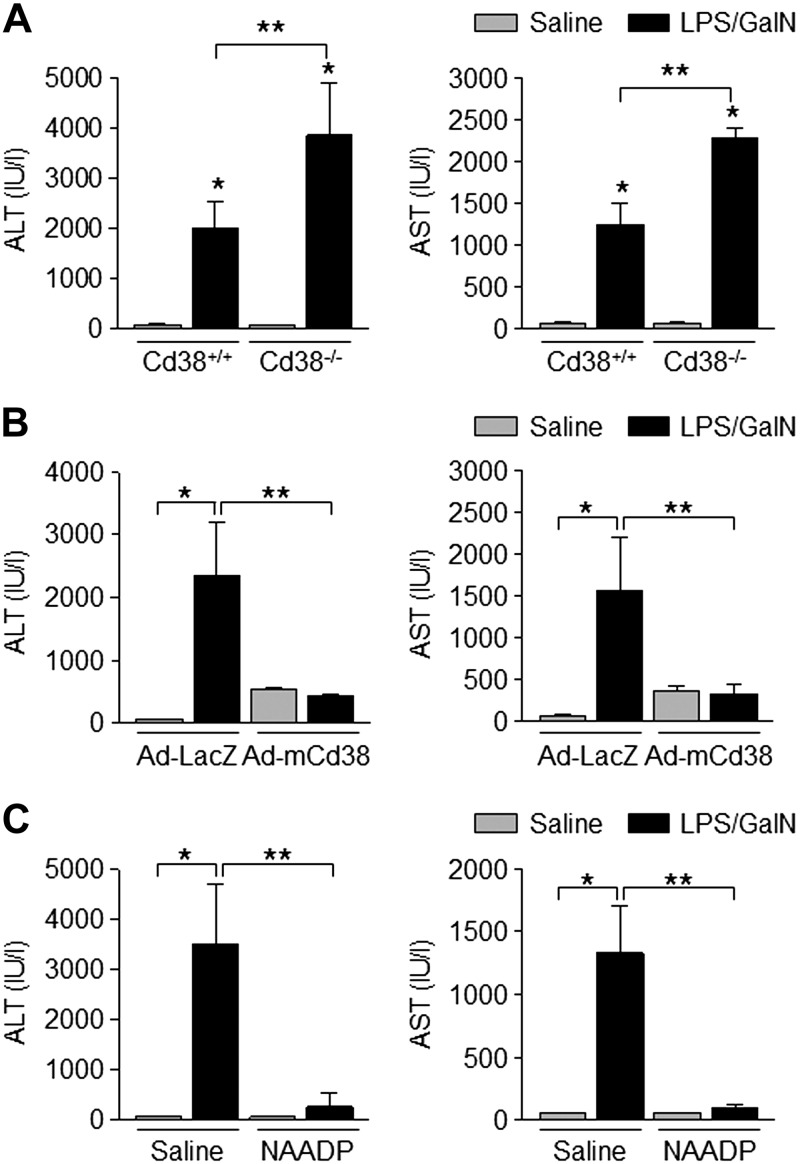
CD38 and NAADP prevent liver injury induced by LPS/GalN. *A*) Serum ALT and AST levels in Cd38^+/+^ and Cd38^−/−^ mice after administration with LPS (2 μg/kg) and GalN (700 mg/kg; *n* = 5). Data are means ± sd. *P < 0.001, **P < 0.05. *B*) Serum ALT and AST levels in mice infected with adenovirus particles (1 × 10^9^ pfu) *via* tail vein 48 h prior to administration with LPS (2 μg/kg) and GalN (700 mg/kg; *n* = 5). Data are means ± sd. *P < 0.001; **P < 0.05. *C*) Serum ALT and AST levels in Cd38^−/−^ mice treated with NAADP (0.181 mg/kg) 30 min after administration with LPS (2 μg/kg) and GalN (700 mg/kg; *n* = 5 or 6). Data are means ± sd. *P < 0.001, **P < 0.05.

Measurements of ALT and AST levels in blood are considered to be important tests to detect liver injury. Here we studied the effect of CD38 and NAADP in protecting the liver from injury induced by LPS/GalN at the whole animal level by measuring ALT and AST in blood. Blood was collected from Cd38*^+/+^* and Cd38*^−/−^* mice before and after LPS/GalN administration, and the levels of ALT and AST in the blood were compared. LPS/GalN increased ALT and AST levels in blood from both Cd38*^+/+^* and Cd38*^−/−^* mice (**Fig. 7*A***). The levels of ALT and AST were greater in Cd38^−/−^ than in Cd38^+/+^ mice.

Similarly, we compared the levels of ALT and AST in the blood collected from mice infected with Ad-mCd38 or Ad-LacZ before and after LPS/GalN administration. LPS/GalN administration produced a strong increase in blood ALT and AST levels in mice infected with LacZ but produced only a weak increase of ALT and AST in mice infected with Ad-mCd38 (Fig. 7*B*).

Finally, we compared ALT and AST levels in the blood from mice intravenously injected with NAADP or saline. As expected, dramatic increases of ALT and AST levels were present in the blood from mice injected with saline. In contrast, mice injected with NAADP showed minimal increases of ALT and AST levels in the blood upon LPS/GalN administration (Fig. 7*C*).

Taken together, our results show that CD38 and NAADP-mediated Ca^2+^ signaling are important mediators for promotion of autophagy, which protects the liver from injury induced by LPS/GalN. Hence, up-regulating the autophagic pathway by CD38/NAADP might be an attractive therapeutic strategy in inflammatory liver diseases.

## DISCUSSION

In the present study, we demonstrate that Cd38*^−/−^* mice administered LPS and GalN showed decreased accumulation of autophagosomes in hepatocytes compared with that in wild-type (Cd38*^+/+^*) mice, suggesting that CD38 is involved in the autophagic process in hepatocytes. The role of CD38 in the autophagy process was further confirmed by finding that CD38 deficiency reduced the expression levels of autophagy-related genes, aggravated hepatic cell death, and elevated the levels of ALT and AST in blood. These results show that autophagy is an important mechanism that protects the liver from inflammatory toxicity.

CD38 is an enzyme that produces NAADP, the most potent intracellular Ca^2+^-mobilizing signaling molecule (32). We found that LPS induced Ca^2+^ increase in Cd38*^+/+^* hepatocytes but not in Cd38*^−/−^* hepatocytes (Fig. 5*A*) and that the Ca^2+^ signals were found to be caused by NAADP (Fig. 5*B*). Moreover, the administration of NAADP to mice reduced the LPS/GalN-induced hepatic injury *via* enhanced autophagy process, suggesting that CD38 and NAADP both promote the autophagy process.

Although the mechanisms by which Ca^2+^ controls autophagy have been controversial (33, 34), mounting evidence indicates that elevated cytosolic Ca^2+^ concentration promotes the autophagic process (13). The molecular mechanisms or regulatory pathways for autophagy in the liver have not been extensively studied. In this study, we unveiled a potential role of CD38 and the Ca^2+^-mobilizing messenger NAADP as an important regulator of the autophagy process in hepatocytes. We provide new insights into the mechanisms by which NAADP-mediated Ca^2+^ signals influence the autophagy processes and protect hepatic injury.

Considering the fact that autophagy represents a catabolic mechanism to degrade proteins and organelles (35), one can postulate that NAADP-mediated Ca^2+^ signaling might result in the degradation of TNF-α by promoting autophagy to protect the liver from LPS/GalN toxicity (36). Many harmful inflammatory cytokines or apoptotic cell death proteins can be produced by TNF and LPS. Alternatively, NAADP might control the production of TNF-dependent inflammatory cytokines or apoptotic death proteins in the hepatocytes upon LPS/GalN stimulation. NAADP-mediated Ca^2+^ signaling might target unidentified target proteins, such as survival factors, to interfere with cytotoxic TNF activity.

Autophagic flux refers to the whole process of autophagy, including autophagosome formation, maturation, fusion with lysosomes, subsequent breakdown, and the release of macromolecules back into the cytosol (2, 3). To assay effects of CD38 and NAADP on autophagic flux, we transfected the Cd38*^+/+^* and Cd38*^−/−^* hepatocytes with the tandem mCherry-GFP-tagged LC3 vector to monitor LC3 puncta formation. LC3 resides in both autophagosomes and autolysosomes, which are recognized in yellow and red, respectively. According to our assay system, there was increased LC3 in both autophagosomes and autolysosomes by CD38 and NAADP, suggesting a role for CD38 and NAADP in promoting autophagic flux.

Our study favors the possibility that NAADP is involved in the early stage of autophagy or autophagosome formation based on 2 lines of evidence. First, the expression of autophagy-related genes was dependent on NAADP-mediated Ca^2^*^+^* mobilization. Second, CD38/NAADP exerted positive effects on both autophagosome and autophagolysosome formation. This is contrast to the reports that CD38 exclusively activates the autophagic flux stage (6). However, we could show a decrease of LPS-induced LC3 expression in Cd38^−/−^ hepatocytes, compared with that in Cd38*^+/+^* hepatocytes, when the autophagosome degradation was blocked by the lysosomal protease inhibitors pepstatin A and E64d (Supplemental Fig. 1), indicating that CD38/NAADP may play a role in autophagosome synthesis under LPS stimulation.

In summary, although the downstream targets for NAADP-mediated Ca^2+^ signaling responsible for the autophagy process have yet to be discovered, the CD38/NAADP signaling pathway elicited the autophagy process, induced the expression of autophagy-related genes, and protected the liver from apoptotic injury induced by LPS. Our results suggest that the survival of the LPS/GalN-treated hepatocytes in this animal experiment results from autophagic promotion by CD38/NAADP. CD38/NAADP-mediated Ca^2+^ signaling pathway is a potential novel therapeutic target against inflammatory liver diseases.

## Abbreviations

Ad-LacZ: LacZ adenovirus
Ad-mCd38: mouse Cd38 adenovirus
ALT: alanine aminotransferase
AST: aspartate aminotransferase
BSA: bovine serum albumin
cADPR: cyclic ADP-ribose
GalN: d-galactosamine
GFP: green fluorescent protein
GPN: Gly-Phe-naphylamide
H&E: hematoxylin and eosin
LC3: mammalian microtubule-associated protein 1 light chain 3
NAADP: nicotinic acid adenine dinucleotide phosphate
TFEB: transcription factor EB
